# Alpha-synuclein spreading in M83 mice brain revealed by detection of pathological α-synuclein by enhanced ELISA

**DOI:** 10.1186/2051-5960-2-29

**Published:** 2014-03-13

**Authors:** Dominique Bétemps, Jérémy Verchère, Sébastien Brot, Eric Morignat, Luc Bousset, Damien Gaillard, Latifa Lakhdar, Ronald Melki, Thierry Baron

**Affiliations:** 1 ANSES - French Agency for Food, Environmental and Occupational Health & Safety, Neurodegenerative Diseases Unit, 31 avenue Tony Garnier, 69364 Lyon cedex 07, France; 2 LEBS, CNRS – Laboratoire d’Enzymologie et Biochimie Structurales, Bâtiment, 34 Avenue de la Terrasse, 91198 Gif-sur-Yvette, France

**Keywords:** Parkinson’s, Dementia, Alpha-synuclein, Prion, ELISA

## Abstract

**Background:**

The accumulation of misfolded proteins appears as a fundamental pathogenic process in human neurodegenerative diseases. In the case of synucleinopathies such as Parkinson’s disease (PD) or dementia with Lewy bodies (DLB), the intraneuronal deposition of aggregated alpha-synuclein (αS) is a major characteristic of the disease, but the molecular basis distinguishing the disease-associated protein (αS^D^) from its normal counterpart remains poorly understood. However, recent research suggests that a prion-like mechanism could be involved in the inter-cellular and inter-molecular propagation of aggregation of the protein within the nervous system.

**Results:**

Our data confirm our previous observations of disease acceleration in a transgenic mouse line (M83) overexpressing a mutated (A53T) form of human αS, following inoculation of either brain extracts from sick M83 mice or fibrillar recombinant αS. A similar phenomenon is observed following a “second passage” in the M83 mouse model, including after stereotactic inoculations into the hippocampus or cerebellum. For further molecular analyses of αS^D^, we designed an ELISA test that identifies αS^D^ specifically in sick mice and in the brain regions targeted by the pathological process in this mouse model. αS^D^ distribution, mainly in the caudal brain regions and spinal cord, overall appears remarkably uniform, whatever the conditions of experimental challenge. In addition to specific detection of αS^D^ immunoreactivity using an antibody against Ser129 phosphorylated αS, similar results were observed in ELISA with several other antibodies against the C-terminal part of αS, including an antibody against non phosphorylated αS. This also indicated consistent immunoreactivity of the murine αS protein specifically in the affected brain regions of sick mice.

**Conclusions:**

Prion-like behaviour in propagation of the disease-associated αS was confirmed with the M83 transgenic mouse model, that could be followed by an ELISA test. The ELISA data question their possible relationship with the conformational differences between the disease-associated αS and its normal counterpart.

## Introduction

Pathological accumulation of misfolded alpha-synuclein (αS) plays a central role in the pathogenesis of synucleinopathies, human neuro-degenerative diseases including Parkinson’s Diseases (PD), dementia with Lewy bodies (DLB), and multiple system atrophy (MSA) [1]. PD is characterized by specific lesions in the brain, with Lewy bodies and Lewy neurites representing deposits of aggregated αS. The crucial role of αS in PD was initially discovered during studies of a few genetic cases of the disease caused by point mutations of the SNCA gene coding for αS, or by its duplication or triplication [2,3], which in the latter case indicated that increased levels of the protein were sufficient to cause the disease.

Considerable interest has recently been shown in the hypothesis that αS aggregation could involve a prion-like mechanism for its propagation, involving self-replication and spreading of a misfolded β-sheet enriched pathogenic conformer derived from the normal protein [4,5]. This was triggered by observations of Lewy bodies and neurites in the embryonic mesencephalic neurons grafted in PD patients’brains, when examined over 10 years following the transplant procedure [6,7]. The stereotypical pattern of lesion progression in PD patients had already been well established by Braak who suggested that PD could be initiated by environmental insults (toxins or pathogens) in the enteric nervous system and/or olfactory bulb before propagating to and within the central nervous system [8]. Recent experimental studies have strongly reinforced this hypothesis and some studies involving *in vitro* models suggest that αS aggregation can spread by axonal transport into the neurons and by cell-to-cell transfer [9].

We previously reported the first *in vivo* experimental evidence that a synucleinopathy could be accelerated by inoculating brain extracts containing a disease-associated αS form in a transgenic mouse model (M83) expressing an A53T mutated human αS protein that is associated with a severe motor impairment occurring during aging of mice [5,10]. The idea that αS aggregation could be triggered or accelerated by intra-cerebral inoculation of aggregated αS was further confirmed in the same M83 mouse model by inoculation with fibrillar recombinant αS or brain extracts from human MSA patients, and also after inoculation of C57Bl/6 wild-type mice with either fibrillar recombinant αS or brain extracts from human DLB patients [4,11-13].

## Results

We previously described the acceleration of a synucleinopathy in a transgenic mouse model (line M83) expressing the A53T mutated human αS protein, when mice were intra-cerebrally inoculated with brain extracts prepared from sick old M83 mice [5]. At the stage of clinical disease, these mice specifically showed accumulation in the brain of insoluble pSer129 αS [5,14], with a typical 4 band pattern detected by Western blot corresponding to monomeric and oligomeric αS forms, ubiquitinated or not [4,12,15].

### Development of an ELISA test for disease-associated α-synuclein (αS^D^) detection

**Figure 1:**
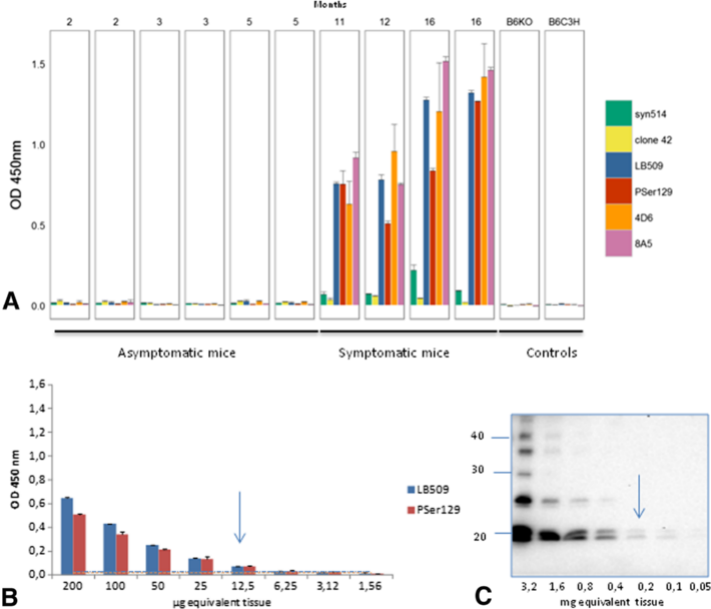
**Detection of disease-associatedα-synuclein (αS**^**D**^**) in M83 mice homogenates from whole brain by ELISA. A**. Anti α-syn antibodies immunoreactivity was tested on 4 symptomatic M83 mice from 11 to 16 months old, in comparison with 6 asymptomatic mice from 2 to 5 months old. B6C3H (genetic background of M83 mice) or B6 αS-null mice were used as additional controls. Sandwich ELISA with rabbit anti-αS polyclonal (capture)/Syn514, LB509, 4D6, 8A5 (reporters) and with clone 42 (capture)/anti-pSer129 αS (PSer129) (reporter) antibodies allows sick mice to be distinguished from asymptomatic M83 mice, whereas ELISA with anti-αS rabbit polyclonal (capture)/clone42 (reporter) does not. Error bars represent S.D. **B**. Determination of the sensitivity of detection of αS^D^ by ELISA. Two-fold dilutions of brain homogenates from a sick mouse were tested, and a positive signal was obtained for 12.5 μg brain equivalents, with both LB509 and PSer129 antibodies. Cut-offs for LB509 and PSer129 were visualized by a line in the color of the antibodies. **C**. Determination of the sensitivity of detection of αS^D^ by Western blot. 200 μg brain equivalents were necessary to detect αS^D^ in Western blot with PSer129 antibody. Molecular weight markers (in kDa) are indicated on the left of the blot.

We have now developed an ELISA test that specifically identifies the disease-associated αS (αS^D^) in brain homogenates prepared in High Salt buffer from sick M83 mice, without any concentration step, unlike Western blot that requires ultracentrifugation in the presence of sarkosyl to detect the protein (Additional file 1: Figure S1B) [14]. Immunoreactivity readily distinguishes old and sick (> 8 months old) from young and healthy (2–5 month old) M83 mice (Figure 1A), using an antibody specifically recognizing the pSer129 αS (p = 0.0074). However it is interesting that several other antibodies showed similarly high immunoreactivities in brain homogenates from sick mice, including 4D6 (p = 0.01), LB509 (p = 0.0047), 8A5 (p < 0.001) against different sequences of the C-terminal part of the protein (124–134, 115–122, and 129–140 respectively) and, to a much lesser extent, Syn514 against the N-terminal end (2–12) of the protein (p = 0.0003). In contrast, under these experimental conditions, analysis with clone 42 reporter antibody, against a central region of α-synuclein (91–96), did not allow to distinguish sick and healthy M83 mice (p = 0.1158). As with other antibodies, a higher imunoreactivity was found in young M83 mice, compared to non transgenic B6C3H mice and still more importantly to B6 αS-null [16] mice, consistently with Western blot analysis of crude brain homogenates (Additional file 1: Figure S1C). As αS^D^ has never been detected in M83 mice younger than 4–6 months [4,5,10,15,17], this likely represents detection of normal human αS overexpressed in M83 mice, which however remains limited under these ELISA conditions.

The sensitivity of this ELISA test for αS^D^ detection was compared to the previously described Western-blot method [5], by using both tests to examine serial dilutions from the same sick M83 mouse brain (Figure 1B). Based on the results obtained during 3–6 repeats of ELISA measures from samples of healthy M83 mice, the estimated cut-off level for discrimination of sick and healthy mice (means + 3 standard deviations) was 0.030 and 0.020 for LB509 and PSer129 antibodies respectively. Under these conditions, a positive ELISA signal was obtained for the brain homogenate from a sick mouse with ~ 10 μg brain equivalents, with both LB509 and PSer129 antibodies, whereas at least 200 μg brain equivalents were necessary to detect pSer129 αS in the sarkosyl insoluble fraction by Western blot with the same PSer129 antibody (Figure 1C).

### Detection of αS^D^ in M83 mice after inoculation of brain extracts from sick mice

**Figure 2:**
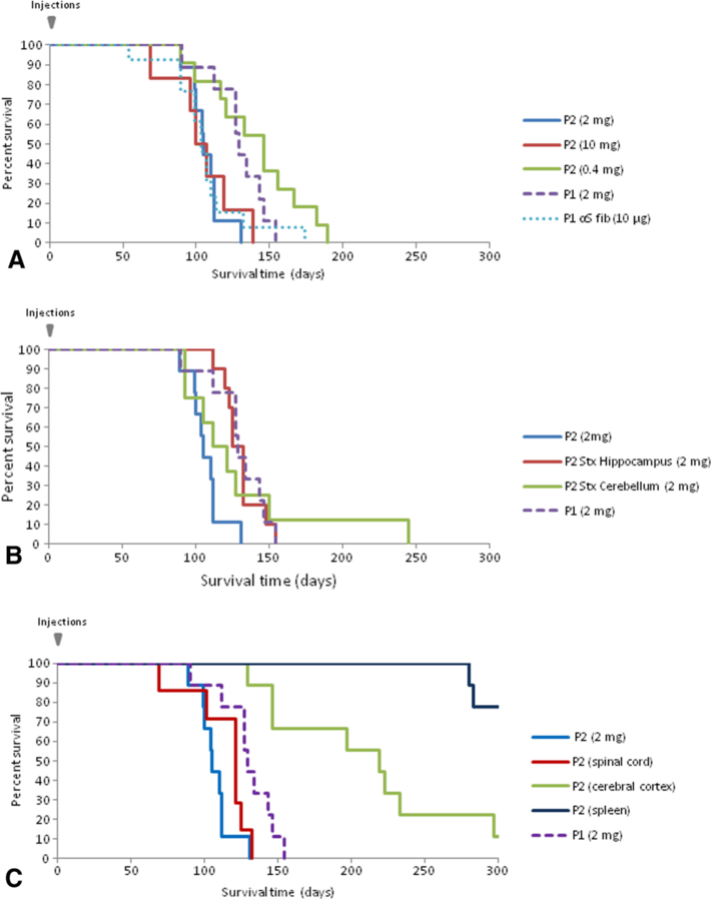
**Survival curves of M83 mice after intracerebral inoculations with brain extracts from sick M83 mice or with fibrillar recombinant protein.** The proportions of surviving mice following inoculations are shown. **A**. Second passage performed with 10 mg (red) (n = 5), 2 mg (blue) (n = 8) and 0.4 mg (green) (n = 10) of brain equivalents, in comparison to a first passage performed with 2 mg (purple-dot) (n = 8) of brain equivalents, or 10 μg of fibrillar recombinant αS. **B**. Second passages performed by stereotactic inoculations with 2 mg brain equivalents from sick M83 mice into the hippocampus (red) (n = 5) or the cerebellum (green) (n = 4), in comparison to a first passage (purple-dot) (n = 8) or a second passage using manual inoculation (blue). **C**. Second passages with 2 mg of spinal cord (red) (n = 9), cerebral cortex (n = 9) (green) or spleen (dark blue) homogenates, in comparison to a first (purple-dot) (n = 8) or second passage (blue) (n = 8) performed with 2 mg of total brain homogenates.

We then examined sick M83 mice that developed early clinical signs following intra-cerebral inoculation of brain extracts from sick M83 mice. This included mice that became sick at an early age (~ 4–6 months old) when inoculated with brain extracts from uninoculated M83 mice which had developed the disease during aging [10] (“first passage”, P1) [14], and also from mice inoculated with brain extracts from these “first passage” mice (“second passage”, P2) (Table 1). The survival period after this second passage was again strikingly reduced in comparison to uninoculated mice, after the inoculation of three different concentrations of brain homogenates (5, 1 or 0.2%, *i.e.* corresponding to 10, 2 or 0.4 mg of brain equivalents per mouse, respectively). Whereas the mice survival periods did not differ significantly for the two largest amounts of brain homogenates injected (2 and 10 mg) (p =0.8592) the animals survived significantly longer when smaller amounts (0.4 mg) of brain homogenates (p = 0.0393) were injected (Figure 2A). Survival periods had also been significantly longer in the previous first passage experiment performed from a 1% homogenate of an old uninoculated and sick mouse (p = 0.0038) [5]. Importantly however, and in contrast with uninoculated M83 mice, all mice receiving brain homogenates that contained aggregated αS developed clinical disease before they were 8 months old.

**Table 1:** List of experiments performed on M83 mice

**Experiment**	**Passage Inoculum (brain equivalent or quantity of recα-syn)**	**Survival period (d.p.i.)**	**Median/maximal survival (days old)**	**αS**^**d**^**detection by Western blot or ELISA or IHC**
1	P1 (2 mg)	129 +/− 19	192/217	8/8
2	αSrec fib (10 μg)	106 +/− 27	145/216	6/6
3	P2 (10 mg)	105 +/− 23	146/181	5/5
4	P2 (2 mg)	107 +/− 12	147/173	8/8
5	P2 (0.4 mg)	140 +/− 33	188/232	9/10
6	P2 Stx hippocampus (2 mg)	130 +/− 13	171/196	5/5
7	P2 Stx cerebellum (2 mg)	131 +/− 50	159/187	4/4
8	P2 spinal cord (2 mg)	113 +/− 22	163/174	5/5
9	P2 cerebral cortex (2 mg)	224 +/− 93	219/472	5/5
10	P2 spleen (2 mg)	378 +/− 98	374/584	4/5

Inoculations were performed at 6 weeks except for experiment 1 (9 weeks).

P1: passage 1, P2: passage 2, Stx: stereotactic, d.p.i.: days post inoculation.

**Figure 3:**
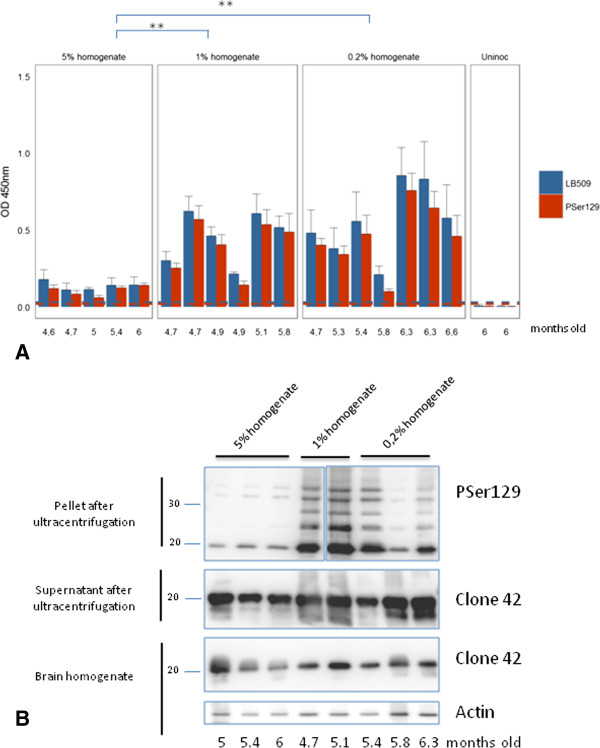
**Detection ofαS by ELISA and Western blot in mice inoculated with different concentrations of whole brain homogenates. A**. Detection by ELISA of αS^D^ with both LB509 and PSer129 antibody in sick M83 mice following inoculation with different concentrations of brain homogenates (5, 1 or 0.2%, P2), and age-matched uninoculated mice. For the 5% brain homogenate, the signal is statistically lower than for the 1% and 0.2% brain homogenates (**, p = 0.01). **B**. Western blot detection of αS with PSer129 antibody in the ultracentrifugation pellets in second passage experiments at different concentrations (experiments 3–5). As observed in ELISA, lower levels of αS^D^ were detected in the pellets prepared from mice inoculated with the 5% brain homogenate, in comparison with most of the mice inoculated with the 1% and 0.2% brain homogenates. In contrast, comparable levels of αS were detected by clone 42 antibody in crude brain homogenates and in the supernatants obtained after ultracentrifugation. Comparable amounts of brain materials were loaded in each lane, as shown by an anti-β-actin antibody used as a loading control. Ages at death of the mice are indicated below the graphs.

ELISA analyses of brain homogenates using an antibody against pSer129 αS also clearly distinguished sick inoculated and healthy uninoculated age-matched M83 mice (Figure 3A). No significant difference in immunoreactivity was observed between inoculated and uninoculated sick M83 mice (p = 0.77). However, as in aged uninoculated mice, the levels of αS^D^ in inoculated M83 mice were variable, and can be roughly divided into two groups of either low (OD < 0.2) or high (OD 0.2 – 1) immunoreactivity. Similar results were obtained with the two antibodies LB509 and PSer129, chosen for further ELISA studies. These individual variations of ELISA immunoreactivities were also quite consistent with those observed by Western blot analysis with the PSer129 antibody of the pellets obtained after ultra-centrifugation from the same mouse brains (Figure 3B). The levels of αS^D^ detected were lower in all mice inoculated with the highest amount of brain extract (10 mg), differing significantly (p = 0.01) from the two other groups of mice inoculated with 2 or 0.4 mg of brain equivalents. The mice inoculated with 10 mg of brain tissue showed concomitantly lower αS^D^ detection (Figure 3) and shorter survivals (Figure 2A).

### Neuro-anatomic distribution of αS^D^ in sick M83 mice

In addition to using some mice from the previously described experimental groups, we also performed experiments (Table 1) including i) inoculation of fibrillar recombinant αS (10 μg/mouse) (experiment 2) and ii) two second passage experiments during which brain extracts from sick M83 mice (2 mg brain equivalents/mouse) were stereotaxically inoculated in the hippocampus (experiment 6) or cerebellum (experiment 7). The M83 mice also exhibited acceleration of the disease with the characteristic motor clinical signs, both in mice inoculated with recombinant fibrillar αS (Figure 2A) and stereotaxically with brain extracts (Figure 2B). Survivals of the animals after inoculations did not significantly differ from that previously observed in the first passage experiment by inoculation of a brain from an old and sick mouse (experiment 1) (p = 0.0979, 0.852 and 0.8205 for experiments 2, 6 and 7 respectively). There was no statistical difference between stereotactic inoculation to hippocampus or cerebellum (p = 0.725).

Also, another second passage experiment using inoculation of a spinal cord homogenate similarly showed an acceleration of disease onset (Figure 2C) with the characteristic motor clinical signs, comparable to that previously observed with whole brain homogenate, whereas inoculation of a cerebral cortex homogenate from the same mouse accelerated the disease to a lesser extent. No reduction in survival time was observed in M83 mice inoculated with a spleen homogenate of this same mouse. Survivals in these three experimental groups (experiments 8–10) significantly differ from each other (p < 0.05).

**Figure 4:**
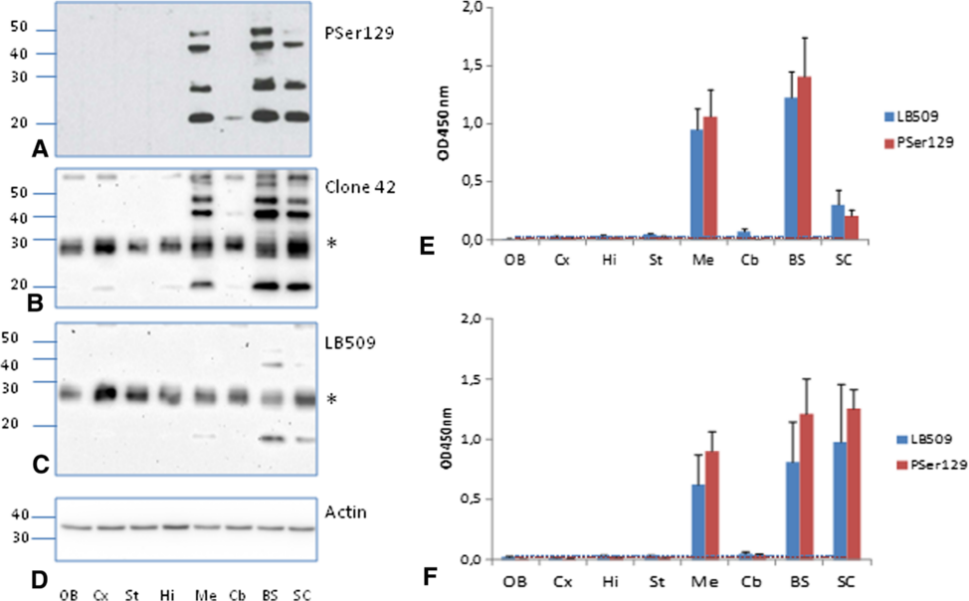
**Detection of disease-associatedα-synuclein (αS**^**D**^**) in different neuro-anatomical regions of M83 mice by ELISA and Western blot.** The following neuro-anatomical regions were sampled from sick M83 mice at first passage: OB: olfactive bulb, Cx: cerebral cortex, Hi: hippocampus, St: striatum, Me: mesencephalon, Cb: cerebellum, BS: brain stem, SC: spinal cord. **A-C**. αS^D^ was identified by Western blot with PSer129 antibody **(A)**, clone 42 **(B)** or LB509 **(C)** antibodies. In **(D)** the blots were revealed by an anti-β-actin antibody as a loading control. **E-F**. ELISA results in these same neuro-anatomical regions were obtained with PSer129 or LB509 antibodies**,** in mice inoculated with brain homogenates from sick M83 mice (P1) (n = 1, 4 repeats, mean ± SD) **(E)** or with fibrillar recombinant αS (n = 4, mean ± SD) **(F)**. Molecular weight markers (in kDa) are indicated on the left of panels **A-D**. Equal amounts of total proteins were used for equivalent loading on gel. *corresponds to a non specific band as shown by the presence of a similar band in the absence of primary antibody and in αS-null mice (data not shown).

Western blot studies after brain dissection revealed insoluble pSer129 αS mainly in the mesencephalon, brain stem and spinal cord, and faintly in the cerebellum (Figures 4A-D), as in uninoculated sick and old M83 mice (data not shown). The same bands were also recognized by clone 42 and, to a lesser extent, LB509 antibodies (Figures 4B-C). As shown for mice inoculated with brain extracts from sick mice at first passage (Figure 4E), or with fibrillar recombinant αS (Figure 4F), brain homogenates from these brain regions (mesencephalon, brain stem and spinal cord) reacted strongly with both LB509 and PSer129 antibodies in ELISA. Other brain regions including the olfactory bulb, cerebral cortex, striatum, hippocampus, thalamus and hypothalamus typically did not show any detectable αS^D^ by Western blot or ELISA. A faint signal in the cerebellum was obtained with both ELISA and Western blot.

**Figure 5:**
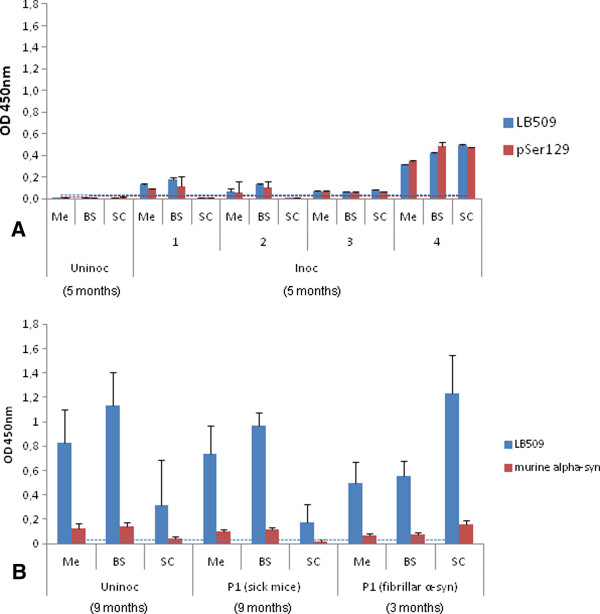
**Species and time-specific detection of disease-associatedα-synuclein (αS**^**D**^**) in selected neuro-anatomical regions.** The following neuro-anatomical regions were sampled from sick M83 mice: Me: mesencephalon, BS: brain stem, SC: spinal cord. **A**. ELISA results for 4 mice (1–4) inoculated (Inoc) with brain extract from sick M83 mice (P2) 12 weeks after the intra-cerebral inoculation, compared to an age-matched uninoculated mouse (Uninoc). **B**. Detection of αS^D^ of mouse origin in sick M83 mice using a mouse specific anti-αS antibody, D37A6, in an uninoculated (Uninoc) sick mouse (276 days old) and in sick M83 mice inoculated with brain extracts from sick mice (P1) or with fibrillar human recombinant αS (231 and 54 days post-inoculation respectively).

The kinetic of αS^D^ detection by ELISA was examined by sacrificing M83 mice 8 or 12 weeks following their inoculation with brain homogenate from a sick M83 mouse (“second passage”). Immunoreactivity was detected in the mesencephalon, brain stem and spinal cord of mice sacrificed at 12 weeks, but not in age-matched uninoculated mice (Figure 5A) or in those sacrificed at 8 weeks (data not shown).

We also examined the possible αS^D^ detection of mouse origin, in sick M83 mice using a mouse specific antibody, D37A6. This revealed immunoreactivity only in the mesencephalon, brain stem and spinal cord of sick mice (Figure 5B). This was similarly observed in uninoculated aged and sick M83 mice and in M83 mice inoculated with brain extracts from sick mice or with fibrillar human recombinant αS. These data suggest that recruitment of the mouse protein occurs within the disease-associated αS aggregates.

**Figure 6:**
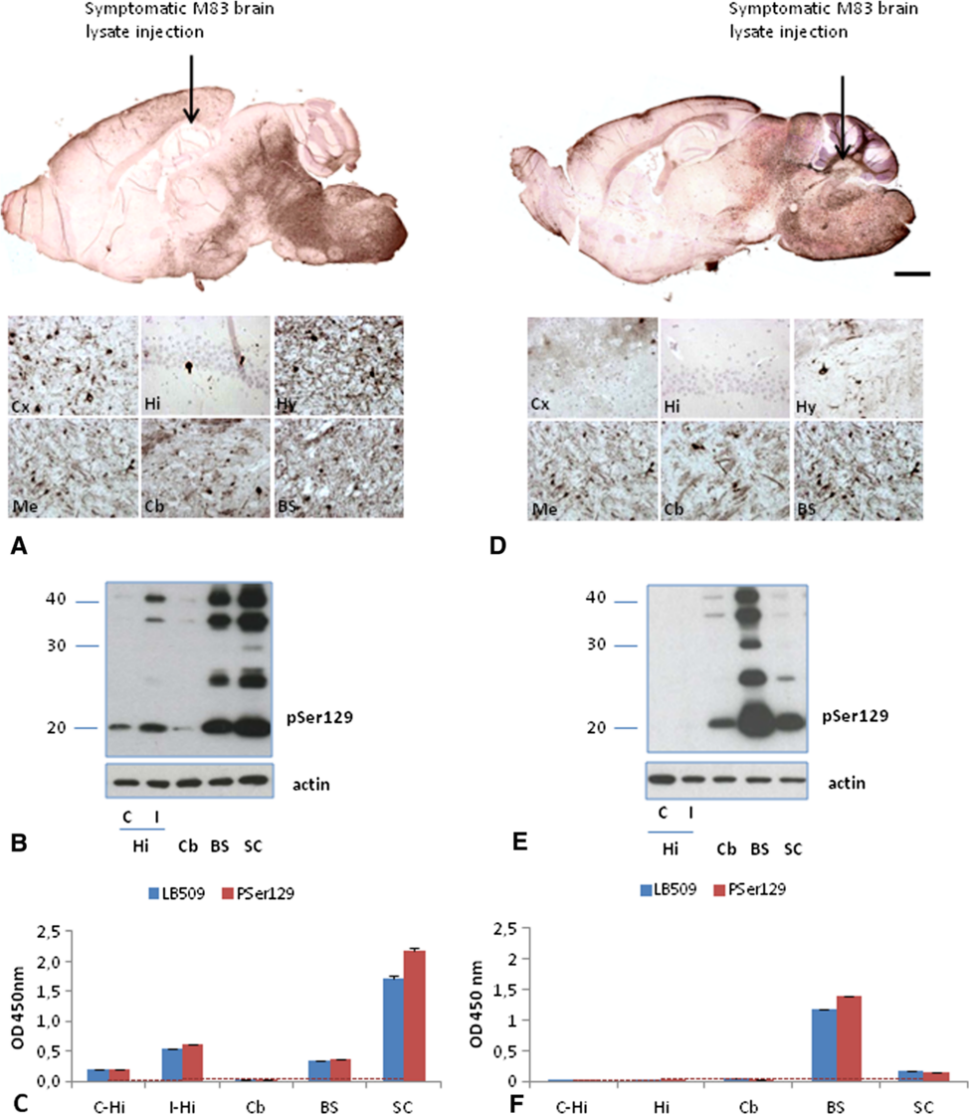
**Detection of disease-associatedα-synuclein (αS**^**D**^**) in different neuroanatomical regions of M83 mice after stereotactic inoculations in the hippocampus (A-C) or cerebellum (D-F) A, D.** Detection of αS on sagittal brain sections by immunohistochemistry using PSer129 antibody. (Scale bar, 1 mm. Insets are high magnifications of different areas). Arrows indicate the approximate sites of the stereotactic inoculations. **B**, **E**. Detection of insoluble pSer129 αS by Western blot in selected neuro-anatomical regions. β-actin was used as loading control. **C**, **F**. ELISA results with LB509 and PSer129 antibody in selected neuro-anatomical regions. Hi: hippocampus (Hi), **C**: contro-lateral side, I: inoculated side, Cb: cerebellum, BS: brainstem, SC: spinal cord.

Results of αS^D^ detection by immunohistochemistry, Western blot and ELISA were then compared in mice stereotaxically inoculated in the hippocampus or cerebellum (second passage) (Figure 6). Immunohistochemistry clearly showed a strong accumulation of pSer129 αS in the caudal regions of the brain, such as the mesencephalon, brain stem and in the spinal cord (Figure 6A and D), but also showed αS^D^ labeling in more frontal brain regions such as the cerebral cortex (insets of Figure 6A and Additional file 1: Figure S1A). The levels of αS^D^ were higher at the inoculation site, *i.e.* in the hippocampus or at the deep cerebellar nuclei. In mice inoculated in the hippocampus, the accumulation of αS^D^ was greater in the inoculated hippocampus (I) than in the contro-lateral side (C) (Figure 6B). αS^D^ labeling was more important in the cerebral cortex following inoculation in the hippocampus than in the cerebellum. In mice inoculated in the cerebellum, no additional reactivity was observed in the cerebellum sample when examined by ELISA, in contrast to Western blot and immunohistochemical analysis. Apart from this, Western blot, ELISA and immunohistochemistry provided very similar results, again emphasizing the stereotyped distribution of αS^D^ more strongly accumulating in the caudal parts of the brain of inoculated mice in this transgenic mouse model [4,10].

## Discussion

Our previous studies showed a striking acceleration of synucleinopathy in a transgenic mouse model (M83) expressing the A53T mutated human αS protein, when mice were intra-cerebrally inoculated with brain extracts from old, sick M83 mice [14]. We have now confirmed these initial observations in experiments using hemi-brain extracts from these mice that showed accelerated disease after experimental challenge, thus representing a “second passage” of the disease, as is routinely performed to propagate prion strains in experimental models. In these experiments, similar incubation periods of the disease were observed after stereotactic inoculation of the brain extracts into either the cerebellum or hippocampus, although this latter brain region is acknowledged to be largely spared during normal aging in this mouse line [4]. All but one mouse inoculated with hemi-brain extracts in the cerebellum developed the disease to a terminal stage well before the age of 8 months, i.e. the age of disease onset in uninoculated M83 mice [10]. Survival, in these second passage experiments, was significantly longer when an extract prepared from the cerebral cortex from a sick mouse was used, with 5/9 mice surviving more than 8 months, although these mice developed the disease more rapidly than uninoculated M83 mice, which is consistent with the much lower levels of αS^D^ in this brain region (Additional file 1: Figure S1A). In the cerebral cortex, αS^D^ is however clearly detected in our study by immunohistochemistry, although to a lesser extent compared to more caudal regions or the spinal cord, consistent with other similar studies [4]. In the second passage experiment performed by non stereotactic inoculation (experiment 4), survival was significantly shorter with 1% whole brain homogenate (from an inoculated mouse) compared to that previously observed at first passage (from an old uninoculated mouse) (experiment 1) [5], although insoluble pSer129 αS^D^ levels in the inocula were comparable (Additional file 1: Figure S1A); in this experiment, a 20 μl volume at 1% of inoculum was inoculated instead of 2 μl at 10% in the stereotactic experiments which showed a similar survival to that observed at first passage. The precise mechanisms and molecular species that contribute to the acceleration of the disease in such experiments are far from being understood yet. A recent study showed induction of αS pathology by intra-cerebral inoculation of a non amyloidogenic form of recombinants αS, raising the question of the contribution of neuroinflammation in such experiments [18]. In addition, the presence of αS oligomers has been described in all examined brain regions of M83 mice, sharing similar basic biochemical properties but showing subtle conformational differences, those found in inclusion-bearing brain regions significantly accelerating αS aggregation *in vitro* and causing primary cortical neuron degeneration [19]. The contribution of these oligomers to the development of neuronal dysfunction appears to be independent of their absolute quantities and basic biochemical properties but is dictated by the composition and conformation of the intermediates as well as unrecognized brain-region-specific intrinsic factors. Such molecular species could differ between first and second passage experiments, as brain lesions appeared more important after inoculation than during normal aging [4].

We then used these experiments to obtain a detailed characterization of αS aggregation in the brain. Western blot analyses specifically revealed pSer129 αS in the insoluble fractions prepared by ultracentrifugation in the presence of sarkosyl, most abundantly in the mesencephalon, brain stem and spinal cord. This disease-associated αS protein (αS^D^) was typically undetected or only barely detectable by this method in the more frontal brain regions, such as the olfactory bulb, cerebral cortex, hippocampus or striatum. The typical 4 band pattern, representing monomeric or oligomeric ubiquitinated or not αS forms [4,12,15], was similarly recognized by clone 42 antibody and, to a much lesser extent, by LB509 antibody. Similar αS^D^ distribution was observed by immunohistochemistry using an antibody against pSer129 αS, which however also revealed specific immunolabeling of neurons in more frontal parts of the brain such as the cerebral cortex. In addition, when stereotaxically inoculated into the hippocampus, αS^D^ was also detected in this brain region, more intensely in the inoculated side of the brain than in the controlateral hippocampus. More intense αS^D^ labeling was also observed in the cerebellum of mice that had been stereotactically injected in this brain region. These data are consistent with previous reports that αS aggregation was increased and/or earlier around the sites of injection of brain homogenates from sick mice [4,12]. The robust detection of αS^D^ in caudal brain regions that do not share direct innervations with the injection site, similar to that observed during normal aging of M83 mice, further supports the hypothesis of αS^D^ trans-synaptic spreading as a possible mode of propagation of αS pathology [4], this transfer being possibly more or less efficient depending on the molecular species (monomers, oligomers or fibril) [20]. In a recent study involving unilateral inoculation of αS preformed fibrils (PFFs) in the hippocampus of transgenic human P301S mutant tau mice, tau inclusions were reported not only in all parts of the hippocampus, including regions that were more rostral and caudal to the injection site, but also in the contralateral hippocampus, and even in the locus coeruleus, a brainstem structure distant from the injection site [21]. Whereas this tau aggregation was triggered to a greater extent by one of two aggregated forms of αS, both failed to induce substantial αS pathology, in contrast with previous studies in wild-type mice [11]. Interestingly, some recent studies of the genesis of infectious prions from recombinant prion proteins (PrP) suggested a new mechanism, designated “deformed templating”, and postulated a required switch from the protein folding pattern of recombinant PrP fibrils to that of the disease-associated PrP to explain the long silent stage before any disease can occur [22]. However, our study in the M83 transgenic mouse model overexpressing A53T mutated human αS, still confirmed an acceleration of the pathological process after intra-cerebral inoculation of fibrillar recombinant human A53T αS, similar to that observed following the inoculation of brain extracts from sick M83 mice. Recent findings obtained following the analysis of presymptomatic versus symptomatic M83 mice suggested that the formation of αS inclusions could be a relatively rapid and probably synchronized process. Indeed, in mice with sparse or moderate levels of αS inclusions, the distribution of aggregates within affected areas of the neuroaxis was diffuse and without clustering of inclusions, whereas none of the presymptomatic mice exhibited abundant αS inclusions [17].

The disease in M83 mice was further characterized by setting up an ELISA test that has been shown to specifically detect αS^D^ directly from mouse brain homogenates. Indeed, ELISA analyses of brain homogenates, prepared from whole brains without any concentration step, readily enabled sick mice to be distinguished from healthy M83 mice, although some variability in αS^D^ levels was apparent between different mice from the same experimental group. In a second passage experiment with different inocula concentrations, mice inoculated with 10 mg of brain tissue equivalents had significantly lower αS^D^ levels than those inoculated with only 2 or 0.4 mg. The biological significance of this variability, if any, remains unexplained, but the ELISA results were tightly correlated with the Western blot analyses of insoluble pSer129 αS. Although such experiments should still be repeated with larger number of animals, it could suggest subtle differences in αS biochemical properties that could be more difficult to extract when higher amounts of aggregated αS have been inoculated. The possible variable effects of different quantities of aggregated αS inoculated on the features of αS aggregates ultimately found in the brain of recipients have been considered in a recent study, emphasizing that different lesions have been observed in different studies involving inoculations of different quantities of aggregated recombinant αS [23]. Alternatively, considering that mice inoculated with the higher αS^D^ concentrations have also received higher amounts of non-αS brain components in our experiments, it should be noticed that it has been suggested that, beside “prion-like” protein self templating, neuroinflammation could be an important event in such experiments, thus possibly modifying the course of the disease according to the inoculation conditions [18,23], giving shorter survival despite lower αS^D^ levels in the brain of M83 mice. In agreement with Western blot analyses, immunoreactivity was specifically identified in the mesencephalon, brain stem and spinal cord by ELISA, but was not detected in the olfactory bulb, cerebral cortex or striatum. Similarly, it was not detected in the hippocampus, except in mice stereotaxically inoculated in this brain region. Although the analytical sensitivity of the ELISA test on brain homogenates appeared to be ~ 20× more sensitive than our previously described Western blot method on brain extracts prepared by ultracentrifugation in the presence of sarkozyl [5], it remained lower at this stage than that of immunohistochemical detection, which also allows the detection of αS^D^ in individual cells in the frontal brain regions. This is consistent with our observation of accelerated disease following intra-cerebral inoculation of a brain extract prepared from the cerebral cortex from a sick M83 mouse, although these mice showed a longer survival than those inoculated with a spinal cord homogenate. These differences in sensitivity were also apparent in ELISA analyses of mice 8 or 12 weeks after their inoculation with brain extracts from sick M83, which were positive at 12 weeks, but negative at 8 weeks. In contrast, data previously obtained from similar experiments in this same M83 model using immunohistochemistry, showed that αS^D^ could be detected as early as 30 days after experimental challenge [4]. All together, our data show clear ELISA differentiation between sick and healthy mice, and between brain regions differently affected by the pathological process, thereby demonstrating that the ELISA approach specifically recognizes disease-associated αS.

Importantly, similar results were obtained by this ELISA test, not only with a monoclonal antibody specifically recognizing pSer129 αS^D^, but also with several other antibodies. It is however remarkable that we failed to detect any immunoreactivity by ELISA analysis of sick M83 mice using the clone 42 antibody against a central region of αS (91–96) [24,25]. This suggests that the recognized epitope could be cryptic under our ELISA conditions while it is exposed in samples denatured for Western blot detection. This agrees with our recent structural investigations which revealed that αS residues 91–93 or 91–97 are involved in beta-sheet structures within aggregated αS [26]. Only very slight immunoreactivity was observed with an antibody against the N-terminal end (2–14) of the protein, Syn-514, that is reported to recognize conformational variants of αS enhanced by the presence of the double mutation E46K/A53T [15,27]. However much higher immunoreactivity was detected with several antibodies directed against the C-terminal part (115–140) of αS: these included LB509, produced against Lewy bodies (amino acid 115–122) and specifically recognizing the human protein, and 8A5 reported to be produced against 129–140 amino-acid and reacting with αS species in Lewy bodies [28]. Interestingly, high immunoreactivity was also observed with the 4D6 antibody, that was recently described to specifically recognize the 124–134 region of the non phosphorylated form of αS at serine residue 129 [29]. This indicates that non phosphorylated αS could also be significantly involved in the aggregation process in this model, even though recent studies have suggested that this phosphorylation occurs in the brain after Lewy bodies formation and more importantly at their periphery [30,31]. Neuronal inclusions not reacting with pSer129 antibodies by immunohistochemistry have already been reported with the M83 model [15], as well as a dramatic increase of total αS in the brain between the ages of 2 and 6 months [32]. In human PD patients, a recent study in which αS levels were assessed by ELISA in the plasma up to 20 years after the initial symptoms, showed a steady increase of total αS as the disease progressed, whereas the pSer129 αS levels remained relatively constant, suggesting that disease progression could be monitored by measuring non phosphorylated αS [30].

Finally, we were able to use the ELISA approach to demonstrate immunoreactivity with an antibody specifically recognizing mouse αS (amino-acids 103–110) from brain homogenates that also reacted with the antibody against pSer 129 αS or with the LB509 antibody that only recognizes human αS. This immunoreactivity with the anti-mouse antibody, although much smaller than that observed with the two other antibodies, was quite consistent. This result is in line with previous observations in similar experiments involving M83 mice, where it was concluded that murine αS might be recruited during human αS aggregation in M83 mice [4]. More recently, aggregation of murine αS was also reported following intra-cerebral inoculation in wild-type mice, not only of fibrillar recombinant mouse αS [11], but also of fibrillar recombinant human αS or brain extracts from human patients with dementia with Lewy bodies [12].

## Conclusions

Our data confirm the consistent acceleration of disease following intra-cerebral inoculation of brain extracts from sick M83 mice or fibrillar recombinant αS, suggesting that disease propagation involves a prion-like mechanism, and further indicating that this can also be observed in serial passages, as routinely performed in experimental models of prion diseases. Detailed αS molecular analyses using an ELISA approach revealed striking differences in immunoreactivity with different antibodies in sick mice, questioning their possible relationship with conformational differences between the disease-associated αS and its normal counterpart. These data will however provide useful tools for further experimental studies of the molecular pathogenesis of human synucleinopathies.

## Materials and methods

### Animal experiments

For this study, we used a transgenic mouse line (M83), that over-expresses the human A53T αS (B6; C3H-Tg[SNCA]83Vle/J, The Jackson laboratory, Bar Harbor, ME) [10] and spontaneously develop a dramatic motor phenotype between 8 and 22 months of age. At the end of their life, M83 mice present characteristic clinical symptoms including weight loss, reduced ambulation, severe motor impairment, prostration, and partial hind limb paralysis with overall stiffness of the hind legs and tail. The mice become unable to get up when placed on their backs. Animal experiments performed in this study are summarized in Table 1.

For non stereotactic experiments, 6–9 week-old homozygous M83 mice were anesthetized by 3% isoflurane inhalation and inoculated intracerebrally in the striato-cortical area with i) 20 μl brain homogenates (0.2, 1 or 5% wt/vol in glucose 5%) obtained from half brains of sick M83 mice as previously described [5] or ii) recombinant fibrillar (10 μg per mouse) human A53T αS [26]. In selected experiments, homogenates were similarly prepared from the spinal cord, cerebral cortex or spleen from a same sick M83 mouse. In the case of stereotactic experiments, 6 week-old homozygous M83 mice were anesthetized with a mixture of 20 mg/kg xylazine/ 60 mg/kg ketamine and inoculated intracerebrally with 2 μl brain homogenates (10% in glucose 5%) in the left hippocampus (anterior-posterior (AP):-2,92; medial-lateral (ML): +3; dorsal-ventral (DV):-3.5), or in the cerebellum (AP: −5.8, ML: +2.25, DV: 3) from the bregma. Controls included uninoculated and asymptomatic M83 mice between 2 and 7 months, sick old M83 mice, B6C3H mice (M83 genetic background line) and C57BL/6S (B6 αS-null) mice, presenting a deletion of the α-syn locus (Harlan, Gannat, France) [16].

The animals were housed and cared for in our approved experimental facilities (n° B 69 387 0801), in accordance with the EC Directive 86/609/EEC and with the Cometh, the National Committee for Ethical Experimentation on Animals (protocol n° 11–0043).

### Extractions of α -synuclein

For biochemical analysis, αS extractions were performed as previously described [5,14]. Briefly, about 20% brain homogenates were prepared in high salt buffer (50 mM Tris–HCl, pH 7.5, 750 mM NaCl, 5 mM EDTA, 1 mM DTT, 1% phosphatase and protease inhibitor cocktails) from half of a brain, using a mechanical homogenizer (grinding balls, Fast prep-FTI20, Thermo). In some of the experiments, dissection was performed immediately after euthanasia, and depending on the quantity of available tissue from each part of the dissected brain or spinal cord, 5, 10 or 20% (wt/vol) homogenates were obtained, on ice with a Dounce borosilicate glass grinder. After centrifugation at 1,000 × g (5 min at +4°C), the supernatants were recovered for ELISA analysis and diluted, depending on the initial concentrations of the homogenates. For analysis of the insoluble αS fraction in Western blot, we used about 200 μl of the supernatants. The supernatants were incubated on ice for 15 min in N-lauroylsarcosyl at a final concentration of 10% before being ultracentrifuged at 465,000 × g (1 hour at +4°C) over a 10% sucrose cushion. The pellets (detergent-insoluble/SDS-soluble fraction) were resuspended in 40 μl of TD4215 denaturing buffer (4% SDS, 2% β-mercaptoethanol, 192 mM glycine, 25 mM Tris, 5% sucrose).

### Western blot analysis

After heat denaturation (5 min at 100°C), the proteins were separated by electrophoresis using 12% SDS-polyacrylamide gels before being electroblotted onto polyvinylidene fluoride membranes (BioRad, Marnes La Coquette, France). αS was probed with either mouse monoclonal antibody (mAb) clone 42 against αS (BD Transduction Laboratories), LB509 against human αS (ref ab27766), rabbit mAb against pSer129 αS (ref ab51253; Abcam, Cambridge, UK) or rabbit polyclonal antibody (pAb) against pSer129 αS (ref ab59264; Abcam, Cambridge, UK) (Table 2). Anti-actin mAb (ref ab8226; Abcam, Cambridge, UK) and anti-βsyn (ref ab76111; Abcam, Cambrigde, UK) were used as controls. The membranes were then incubated with horseradish peroxidase conjugated goat anti-mouse (ref 32460; Thermo) or anti-rabbit (ref 32430; Thermo) Ig secondary antibodies (1:1000). The immunocomplexes were visualized with chemiluminescent reagents (Supersignal WestDura, ref 34076, Thermo), followed by exposure on Biomax MR Kodak films, or CL-Exposure films, and by analysis using the Versa Doc system and Quantity One software (both from BioRad).

**Table 2:** Antibodies used in this study

***Antibodies***	**Epitopic specificity**	***Type***	***Source***	***Dilution (ELISA/WB/IHC)***
LB509 (human α-syn)	115-122	Mouse monoclonal	Abcam (ref ab27766)	1/2000^E^ -1/1000^WB^
4D6 (α-syn)	124–134	Mouse monoclonal	Abcam (ref ab1903)	1/2000^E^
clone42 (α-syn)	91–96	Mouse monoclonal	BD Biosciences (ref 610787)	1/2000^E/WB^
syn514 (α-syn)	2–12	Mouse monoclonal	Abcam (ref ab24717)	1/500^E^
8A5 (α-syn)	129–140	Mouse monoclonal	Provided by Dr. Anderson	1/2000^E^
D37A6 (mouse α-syn)	103–110	Rabbit polyclonal	Cell Signaling (ref 4179)	1/1000^E^
phosphorylated α-syn*	PSer129	Rabbit polyclonal	Abcam (ref ab59264)	1/3000^E^ -1/1000^WB^
EP1536Y (phosphorylated α-syn)**	PSer129	Rabbit monoclonal	Abcam (ref ab51253)	1/1000^WB^-1/300^IHC^
EP1537Y (β-syn)		Rabbit monoclonal	Abcam (ref ab76111)	1/5000^WB^
Actin		Mouse monoclonal	Abcam (ref ab8226)	1/2000^WB^

All antibodies were used in ELISA with polyclonal anti α-syn antibody (Millipore ref AB5038P) as capture, unless mentioned.

*Antibody used in ELISA with monoclonal anti α-syn clone 42 as capture.

**Antibody used in Western blot and immunochemistry.

E: ELISA, WB: Western blot.

### Immunohistochemistry

Immunohistochemistry was performed on buffered 10% formalin-fixed samples as previously described, including pretreatments with boiling 5 min in 0.1 mol/L of citrate buffer (pH 6.2) using a microwave oven and 20 min immersion in a 3 mol/L guanidine isothiocyanate solution [5,14]. The primary antibody used was rabbit mAb against pSer129 αS (ref ab51253; Abcam, Cambridge, UK) (Table 2). The secondary antibody was HRP-labeled anti-rabbit Ig antibody (1:200) (ref 401004; Southern Biotech). Antibody binding was detected using the avidin-biotin complex system (Vector Laboratories, Peterborough, UK) revealed by black deposition of diaminobenzidine intensified with nickel chloride.

### ELISA

The αS levels in brain extracts were measured using a sandwich ELISA. Plates (MaxiSorp™, Thermo Scientific Nunc) were coated with capture antibodies, either 0.01 ng/ml anti αS rabbit polyclonal (Millipore, ref AB5038P) or monoclonal clone 42 antibody (BD Biosciences, ref 610787) in 50 mM Na_2_CO3/NaHCO3 (pH9.6) with 100 μl per well at 4°C overnight. Plates were washed 5 times in phosphate-buffered saline with 0.05% Tween20 (PBST). Superblock T20 PBS blocking buffer (Pierce) was then added for 1 h at room temperature (RT) with shaking at 150 rpm. The plates were again washed 5 times in PBST and the brain homogenates (dilution 1:100 of the 20% homogenates in PBST BSA 1%) and standards (human α-syn recombinant-Sigma) were incubated at 25°C for 2 h with shaking. After washing 5 times with PBST, captured αS protein was detected by different antibodies against αS (Table 2). The plates were washed 5 times in PBST and either anti-mouse (ref 1010–05, Southern Biotech) or anti-rabbit (ref 4010–05, Southern Biotech) IgG HRP conjugates was added at 1:8000 dilution in PBST BSA 1% for 1 h at RT. After washing the plates 5 times with PBST, 100 μl of 3,3′,5,5′-tetramethylbenzidine (TMB) solution (ref T0440, Sigma) was added to each well and incubated for 15 min with shaking. The reaction was stopped with 100 μl of 1 N HCl, and the absorbance was measured at 450 nm with the microplate reader Model 680 (Biorad). For data analysis, the OD value obtained in the well with all the reagents except the sample (Blank well) was subtracted from the OD value of each analyzed sample.

### Statistical analysis

The incubation period is the time from the day of inoculation until death (d.p.i.). The distributions of incubation period between groups were compared after fitting the data with Cox regression models. Tests and diagnostics of the proportional hazards assumption of the Cox models were based on weighted residuals. When comparisons were required between several groups, Tukey’s multiple tests method was applied.

Mixed-effects regressions were used to model OD. A mixed effect was used to reflect the variability of the repetitions for a given mouse. Given that the residuals of the models gave a clear indication of heteroscedasticity, the variance matrix was appropriately parameterized. Validation of the model was based on examination of the residuals. Analyses were done with R version 1.15.1 and the packages nlme and survival. A significance threshold of P = 0.05 was used for all experiments.

### Recombinant αS purification and assembly

Recombinant human A53T αS was expressed and purified as described previously [33]. Soluble A53T αS was incubated in buffer A (50 mM Tris–HCl, pH 7.5, 150 mM KCl) at 37°C under continuous shaking in an Eppendorf Thermomixer set at 600 r.p.m. Assembly was monitored continuously using Thioflavin T binding and the nature of the fibrillar assemblies was assessed by electron microscopy as described [34].

## Competing interests

The authors declare that they have no competing interests.

## Authors’ contributions

DB participated in the design of the study, carried out the biochemical experiments, analysed the data, and drafted the paper. JV carried out and analyzed the biochemical experiments. SB carried out and analysed the stereotactic experiments and immunohistochemical analyses. EM performed the statistical analyses. DG participated in the stereotactic experiments and Latifa Lakhdar supervised the animal experiments. LB and RM produced and characterized the fibrillar recombinant protein. TB conceived the study, analyzed the data and drafted the paper. All authors read and approved the final manuscript.

## Supplementary Material

Additional file 1: Figure S1 Western blot detection of αS in asymptomatic versus symptomatic M83 mice and in the inocula used in the study. A. Comparison of levels of αS^D^ in the different inocula used for experiments 1 to 10 (Table 1). αS^D^ was detected in the ultracentrifugation pellets used as inocula for first passage (experiment 1) (P1) or for second passage, from half-brain (P2) (experiments 3–7) or from different areas including spinal cord (SC), cortex (Cx), or spleen (Spl) (experiments 8–10) using PSer129 antibody. **B.** Western blot detection of αS in 20% crude brain homogenates of symptomatic or asymptomatic M83 mice, in comparison to the pellets and supernatants obtained after utltracentrifugation. pSer129 αS was detected only in the pellets of symptomatic mice using PSer129 αS antibody. **C.** Detection of αS in 20% crude brain homogenates was comparable in asymptomatic and symptomatic M83 mice with both clone 42 and LB509 antibodies. No αS was observed in B6 αS-null mice with the same antibodies, and in B6C3H mice also with LB509 antibody that recognizes only human αS. All mice except B6 αS-null mice presented αS detected by D37A6 antibody, specifically directed against murine αS. As a control, β-synuclein was detected in all the mice with the β-synuclein specific antibody EP1537Y [35]. Molecular weight markers (in kDa) are indicated on the left of panels **A-B**. The blots were also revealed by an anti-β-actin antibody as a loading control. Click here for file

## References

[B1] Goedert M Spillantini MG Del Tredici K Braak H 100 years of Lewy pathology Nat Rev Neurol 2013 2 13 24 23183883 10.1038/nrneurol.2012.242

[B2] Farrer M Kachergus J Forno L Lincoln S Wang DS Hulihan M Maraganore D Gwinn-Hardy K Wszolek Z Dickson D Langston JW Comparison of kindreds with parkinsonism and alpha-synuclein genomic multiplications Ann Neurol 2004 2 174 179 10.1002/ana.10846 14755720

[B3] Polymeropoulos MH Lavedan C Leroy E Ide SE Dehejia A Dutra A Pike B Root H Rubenstein J Boyer R Stenroos ES Chandrasekharappa S Athanassiadou A Papapetropoulos T Johnson WG Lazzarini AM Duvoisin RC Di Iorio G Golbe LI Nussbaum RL Mutation in the alpha-synuclein gene identified in families with Parkinson’s disease Science 1997 2 2045 2047 10.1126/science.276.5321.2045 9197268

[B4] Luk KC Kehm VM Zhang B O’Brien P Trojanowski JQ Lee VM Intracerebral inoculation of pathological alpha-synuclein initiates a rapidly progressive neurodegenerative alpha-synucleinopathy in mice J Exp Med 2012 2 975 986 10.1084/jem.20112457 22508839 PMC3348112

[B5] Mougenot AL Nicot S Bencsik A Morignat E Verchere J Lakhdar L Legastelois S Baron T Prion-like acceleration of a synucleinopathy in a transgenic mouse model Neurobiol Aging 2012 2 2225 2228 10.1016/j.neurobiolaging.2011.06.022 21813214

[B6] Kordower JH Chu Y Hauser RA Freeman TB Olanow CW Lewy body-like pathology in long-term embryonic nigral transplants in Parkinson’s disease Nat Med 2008 2 504 506 10.1038/nm1747 18391962

[B7] Li JY Englund E Holton JL Soulet D Hagell P Lees AJ Lashley T Quinn NP Rehncrona S Bjorklund A Widner H Revesz T Lindvall O Brundin P Lewy bodies in grafted neurons in subjects with Parkinson’s disease suggest host-to-graft disease propagation Nat Med 2008 2 501 503 10.1038/nm1746 18391963

[B8] Braak H Rub U Gai WP Del Tredici K Idiopathic Parkinson’s disease: possible routes by which vulnerable neuronal types may be subject to neuroinvasion by an unknown pathogen J Neural Transm 2003 2 517 536 10.1007/s00702-002-0808-2 12721813

[B9] Desplats P Lee HJ Bae EJ Patrick C Rockenstein E Crews L Spencer B Masliah E Lee SJ Inclusion formation and neuronal cell death through neuron-to-neuron transmission of alpha-synuclein Proc Natl Acad Sci USA 2009 2 13010 13015 10.1073/pnas.0903691106 19651612 PMC2722313

[B10] Giasson BI Duda JE Quinn SM Zhang B Trojanowski JQ Lee VM Neuronal alpha-synucleinopathy with severe movement disorder in mice expressing A53T human alpha-synuclein Neuron 2002 2 521 533 10.1016/S0896-6273(02)00682-7 12062037

[B11] Luk KC Kehm V Carroll J Zhang B O’Brien P Trojanowski JQ Lee VM Pathological alpha-synuclein transmission initiates Parkinson-like neurodegeneration in nontransgenic mice Science 2012 2 949 953 10.1126/science.1227157 23161999 PMC3552321

[B12] Masuda-Suzukake M Nonaka T Hosokawa M Oikawa T Arai T Akiyama H Mann DM Hasegawa M Prion-like spreading of pathological alpha-synuclein in brain Brain 2013 2 1128 1138 10.1093/brain/awt037 23466394 PMC3613715

[B13] Watts JC Giles K Oehler A Middleton L Dexter DT Gentleman SM Dearmond SJ Prusiner SB Transmission of multiple system atrophy prions to transgenic mice P Natl Acad Sci 2013 2 19555 19560 10.1073/pnas.1318268110 PMC3845125 24218576

[B14] Mougenot AL Bencsik A Nicot S Vulin J Morignat E Verchère J Bétemps D Lakhdar L Legastelois S Baron T Transmission of prion strains in a transgenic mouse models overexpressing human A53T mutated alpha-synuclein J Neuropathol Exp Neurol 2011 2 377 385 10.1097/NEN.0b013e318217d95f 21487306

[B15] Waxman EA Giasson BI Specificity and regulation of casein kinase-mediated phosphorylation of alpha-synuclein J Neuropathol Exp Neurol 2008 2 402 416 10.1097/NEN.0b013e3186fc995 18451726 PMC2930078

[B16] Specht CG Schoepfer R Deletion of the alpha-synuclein locus in a subpopulation of C57BL/6 J inbred mice BMC Neurosci 2001 2 11 10.1186/1471-2202-2-11 11591219 PMC57740

[B17] Emmer KL Waxman EA Covy JP Giasson BI E46K human alpha-synuclein transgenic mice develop Lewy-like and tau pathology associated with age-dependent, detrimental motor impairment J Biol Chem 2011 2 35104 35118 10.1074/jbc.M111.247965 21846727 PMC3186371

[B18] Sacino AN Brooks M McGarvey NH McKinney AB Thomas MA Levites Y Ran Y Golde TE Giasson BI Induction of CNS alpha-synuclein pathology by fibrillar and non-amyloidogenic recombinant alpha-synuclein Acta Neuropathol Commun 2013 2 38 10.1186/2051-5960-1-38 24252149 PMC3893388

[B19] Tsika E Moysidou M Guo J Cushman M Gannon P Sandaltzopoulos R Giasson BI Krainc D Ischiropoulos H Mazzulli JR Distinct region-specific alpha-synuclein oligomers in A53T transgenic mice: implications for neurodegeneration J Neurosci 2010 2 3409 3418 10.1523/JNEUROSCI.4977-09.2010 20203200 PMC2844128

[B20] Rey NL Petit GH Bousset L Melki R Brundin P Transfer of human alpha-synuclein from the olfactory bulb to interconnected brain regions in mice Acta Neuropathol 2013 2 555 573 10.1007/s00401-013-1160-3 23925565 PMC3789892

[B21] Guo JL Covell DJ Daniels JP Iba M Stieber A Zhang B Riddle DM Kwong LK Xu Y Trojanowski JQ Lee VM Distinct alpha-synuclein strains differentially promote Tau inclusions in neurons Cell 2013 2 103 117 10.1016/j.cell.2013.05.057 23827677 PMC3820001

[B22] Makarava N Kovacs GG Savtchenko R Alexeeva I Budka H Rohwer RG Baskakov IV Genesis of mammalian prions: from non-infectious amyloid fibrils to a transmissible prion disease PLoS Pathog 2011 2 e1002419 10.1371/journal.ppat.1002419 22144901 PMC3228811

[B23] Recasens A Dehay B Bove J Carballo-Carbajal I Dovero S Perez A Fernagut PO Blesa J Parent A Perier C Fariñas I Obeso JA Bezard E Vila M Lewy body extracts from parkinson’s disease brains trigger alpha-synuclein pathology and neurodegeneration in mice and monkeys Ann Neurol 2013 doi:10.1002/ana.24066 10.1002/ana.24066 24243558

[B24] Perrin RJ Payton JE Barnett DH Wraight CL Woods WS Ye L George JM Epitope mapping and specificity of the anti-alpha-synuclein monoclonal antibody Syn-1 in mouse brain and cultured cell lines Neurosci Lett 2003 2 133 135 10.1016/S0304-3940(03)00781-X 12946570

[B25] Emmanouilidou E Elenis D Papasilekas T Stranjalis G Gerozissis K Ioannou PC Vekrellis K Assessment of alpha-synuclein secretion in mouse and human brain parenchyma PLoS One 2011 2 e22225 10.1371/journal.pone.0022225 21779395 PMC3136497

[B26] Bousset L Pieri L Ruiz-Arlandis G Gath J Jensen PH Habenstein B Madiona K Olieric V Bockmann A Meier BH Melki R Structural and functional characterization of two alpha-synuclein strains Nat Commun 2013 2 2575 24108358 10.1038/ncomms3575 PMC3826637

[B27] Duda JE Giasson BI Mabon ME Lee VM Trojanowski JQ Novel antibodies to synuclein show abundant striatal pathology in Lewy body diseases Ann Neurol 2002 2 205 210 10.1002/ana.10279 12210791

[B28] Anderson JP Walker DE Goldstein JM de Laat R Banducci K Caccavello RJ Barbour R Huang J Kling K Lee M Diep L Keim PS Shen X Chataway T Schlossmacher MG Seubert P Schenk D Sinha S Gai WP Chilcote TJ Phosphorylation of Ser-129 is the dominant pathological modification of alpha-synuclein in familial and sporadic Lewy body disease J Biol Chem 2006 2 29739 29752 10.1074/jbc.M600933200 16847063

[B29] Lee BR Matsuo Y Cashikar AG Kamitani T Role of Ser129 phosphorylation of alpha-synuclein in melanoma cells J Cell Sci 2013 2 696 704 10.1242/jcs.122093 23203798 PMC3613186

[B30] Foulds PG Diggle P Mitchell JD Parker A Hasegawa M Masuda-Suzukake M Mann DM Allsop D A longitudinal study on alpha-synuclein in blood plasma as a biomarker for Parkinson’s disease Sci Rep 2013 2 2540 23985836 10.1038/srep02540 PMC3756331

[B31] Waxman EA Giasson BI Characterization of kinases involved in the phosphorylation of aggregated alpha-synuclein J Neurosci Res 2011 2 231 247 10.1002/jnr.22537 21162130 PMC4484797

[B32] Paumier KL Sukoff Rizzo SJ Berger Z Chen Y Gonzales C Kaftan E Li L Lotarski S Monaghan M Shen W Stolyar P Vasilyev D Zaleska M Hirst DW Dunlop J Behavioral characterization of A53T mice reveals early and late stage deficits related to parkinson’s disease PLoS One 2013 2 e70274 10.1371/journal.pone.0070274 23936403 PMC3731353

[B33] Ghee M Melki R Michot N Mallet J PA700, the regulatory complex of the 26S proteasome, interferes with alpha-synuclein assembly FEBS J 2005 2 4023 4033 10.1111/j.1742-4658.2005.04776.x 16098186

[B34] Pieri L Madiona K Bousset L Melki R Fibrillar alpha-synuclein and huntingtin exon 1 assemblies are toxic to the cells Biophys J 2012 2 2894 2905 10.1016/j.bpj.2012.04.050 22735540 PMC3379023

[B35] Newman AJ Selkoe D Dettmer U A new method for quantitative immunoblotting of endogenous alpha-synuclein PLoS One 2013 2 e81314 10.1371/journal.pone.0081314 24278419 PMC3835431

