# Supplementary figures and images for: Alpha-synuclein spreading in M83 mice brain revealed by detection of pathological α-synuclein by enhanced ELISA
Source: Acta Neuropathol Commun. 2014 Mar 13;2:29. doi: 10.1186/2051-5960-2-29 (PMC4007641; doi:10.1186/2051-5960-2-29)

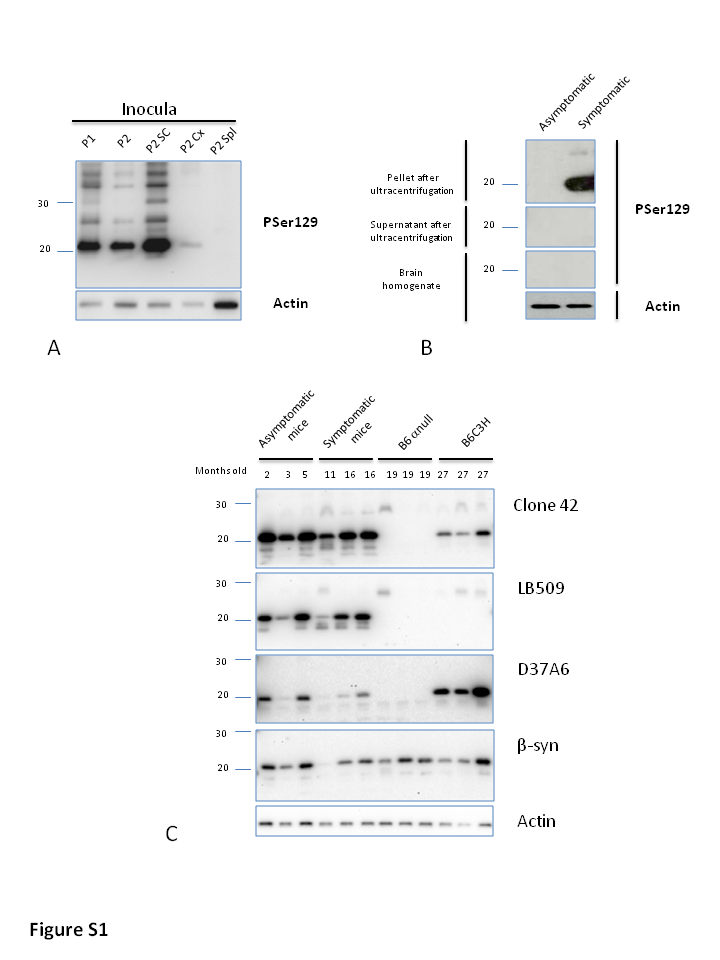

Supplement: Additional file 1: Figure S1 — Western blot detection of αS in asymptomatic versus symptomatic M83 mice and in the inocula used in the study. A. Comparison of levels of αSD in the different inocula used for experiments 1 to 10 (Table 1). αSD was detected in the ultracentrifugation pellets used as inocula for first passage (experiment 1) (P1) or for second passage, from half-brain (P2) (experiments 3–7) or from different areas including spinal cord (SC), cortex (Cx), or spleen (Spl) (experiments 8–10) using PSer129 antibody. B. Western blot detection of αS in 20% crude brain homogenates of symptomatic or asymptomatic M83 mice, in comparison to the pellets and supernatants obtained after utltracentrifugation. pSer129 αS was detected only in the pellets of symptomatic mice using PSer129 αS antibody. C. Detection of αS in 20% crude brain homogenates was comparable in asymptomatic and symptomatic M83 mice with both clone 42 and LB509 antibodies. No αS was observed in B6 αS-null mice with the same antibodies, and in B6C3H mice also with LB509 antibody that recognizes only human αS. All mice except B6 αS-null mice presented αS detected by D37A6 antibody, specifically directed against murine αS. As a control, β-synuclein was detected in all the mice with the β-synuclein specific antibody EP1537Y [35]. Molecular weight markers (in kDa) are indicated on the left of panels A-B. The blots were also revealed by an anti-β-actin antibody as a loading control. [file 2051-5960-2-29-S1.TIF]
